# Structural and Functional Changes of Hands and Legs in Early Rheumatoid Arthritis

**DOI:** 10.3390/medicina57040317

**Published:** 2021-03-28

**Authors:** Annika Valner, Ülle Kirsimägi, Raili Müller, Mart Kull, Kaja Põlluste, Margus Lember, Riina Kallikorm

**Affiliations:** 1 Institute of Clinical Medicine, University of Tartu, L. Puusepa 8, 51014 Tartu, Estonia; ulle.kirsimagi@ut.ee (Ü.K.); raili.muller@kliinikum.ee (R.M.); kaja.polluste@ut.ee (K.P.); margus.lember@ut.ee (M.L.); riina.kallikorm@ut.ee (R.K.); 2 Internal Medicine Clinic, Tartu University Hospital, L. Puusepa 8, 51014 Tartu, Estonia; 3 Viljandi County Hospital, 71024 Viljandi Maakond, Estonia; mart.kull@gmail.com

**Keywords:** early rheumatoid arthritis, muscle, muscle strength, bone, lifestyle

## Abstract

*Background and Objectives:* The aim of this study was to assess if there are structural and functional changes of hands and legs already in early rheumatoid arthritis (ERA), compared with the population-based control group. Additionally, we aimed to identify if the changes are symmetrical in hands and legs and if there are factors that are associated with these changes. The study was conducted, and, thus far, the results have been controversial. *Materials and Methods:* The study group consisted of 83 consecutive patients with ERA and 321 control subjects. Dual-Energy X-Ray Absorptiometry (DXA) machine was used to measure bone, lean and fat mass. Inflammation and bone markers, smoking and nutritional habits were assessed, to evaluate the effects of different factors. The 30-Second Chair Stand Test (30-CST) and the Handgrip Strength Test (HST) were used to estimate muscle strength. *Results:* The presence of ERA was associated with lower arm, leg lean mass and higher fat mass of arm, compared with control subjects. ERA was also associated with lower mean handgrip in HST and worse muscle strength of legs in the 30-CST. Bone mass changes were not so evident both in arms and legs. Smoking habits did not seem to have relevant effect on bone mass, muscle structural and functional changes, both on hands and legs. In ERA, lean mass of arm and leg was negatively associated with C-reactive protein (CRP). The intake of proteins in ERA was not associated with lean mass changes both in hands and legs. *Conclusions:* Structural and functional changes of hands and legs are different in ERA. ERA patients had higher fat mass of arm, lower lean mass of arm and leg and, accordingly, decreased muscle function. The lowering of lean mass of arm and leg in ERA was associated with the elevation of CRP.

## 1. Introduction

Rheumatoid arthritis (RA) is a chronic autoimmune disease mainly affecting the small joints of the hands and feet. It is characterized by persistent synovitis, accompanied with systemic inflammation [1], the destruction of cartilage and underlying bone [2], and sometimes the presence of autoantibodies (rheumatoid factor (RF) and anti-citrullinated protein antibodies (anti-CCP)) [1]. RA affects 0.5–1.0% of adults, with 5–50 per 100,000 new cases annually, and is most common in women and elderly people. The prevalence of RA in Estonia, among women and men 20 years and older, was 0.70% (6.68–7.37) and 0.16% (1.42, 1.79) [3]. The main environmental risk factor is smoking, and 50% of the risk of developing RA may be related to genetic factors [1]. Smoking is a risk factor for RA, especially among RF-positive RA men and heavy smokers [4,5]. Smoking is also known to trigger Human Leukocyte Antigen-DR isotype (HLA-DR) restricted immune reactions to autoantigens modified by citrullination [5]. Anti-CCP positive RA is regarded as more severe [6] disease subset, and this antibody can possibly mediate the effect of smoking on joint damage in RA [5,7].

An early characteristic sign of appendicular bone damage is peri-articular osteoporosis or osteopenia that precedes the development of erosions [8]. Dual Energy X-ray Absorptiometry (DXA) is a widely used sensitive method to evaluate bone mass, and in the case of ERA, can be a useful tool in predicting radiographic progression or functional status [8,9,10]. In addition to local bone changes in RA, systemic bone loss can appear [11]. Hand bone loss may already occur in the early phase of RA [12,13]. Current knowledge about leg bone mass changes due to ERA is insufficient. Even though treatments have become more available and effective, 70% of patients still develop RA specific bone structure changes in the three first years of disease [14]. A total of 25% of patients already experience them within three months of developing RA [15]. DXA is usually conducted to evaluate bone mass, but it also enables to assess muscle and fat mass of hands and legs in parallel.

Local and generalized bone loss have the same pathway: the receptor activator of nuclear factor kappa-B ligand (RANKL)/osteoprotegerin (OPG) route [2,16], which stimulates the activation, differentiation and proliferation of osteoclasts [2,17]. Several inflammatory cytokines (TNF-α, interleukin (IL)-1, IL-6 and IL-17) upregulate RANKL and subsequently activate osteoclastogenesis [2,18,19]. RANKL acts as a critical factor in the appearance of joint destruction in RA [2]. OPG is thought to inhibit bone damage following synovial inflammation [11] and may originate from body fat [20,21]. Androgens inhibit OPG levels, while estradiol has an opposite effect [20,22]. Immobilization contributes to bone loss, and in the case of RA, mechanical loading of bone may prevent osteoclast-related bone loss [23].

A recent study showed that ERA patients had lower appendicular lean mass compared to healthy subjects, an effect related to catabolism-inducing factors (inflammation) and lower protein intake [24]. In addition, muscle strength had a crucial role in preventing arthritic bone loss [25].

In RA, usually only the small joints of hands are evaluated when assessing disease activity, as most widely used scales do not assess the small joints of legs. Still, half of RA patients already experience foot problems by the time of diagnosis [26,27], and in a third of cases, foot pain was the prevalent symptom [26,28] affecting everyday life and coping [26,29]. Arthritis involvement may be different among people. Therefore, it is important in ERA to assess hands and legs in parallel, as this may alter the evaluation of disease activity and thus affect treatment strategy.

The study was conducted, as, thus far, the results have been controversial. The aim of this study was to assess if there are structural and functional changes of hands and legs already in early RA, compared with the population-based control group. Additionally, we aimed to identify if the changes are symmetrical in hands and legs and if there are factors that associate with these changes.

## 2. Materials and Methods

The study group consisted of 83 patients with ERA (age: 19–79 years) and 321 subjects in a control group (age: 20–79 years). One hundred consecutive patients referred to Tartu University Hospital with newly diagnosed RA were recruited between 2012 and 2014, in order to form the ERA group. Their diagnosis was established according to American College of Rheumatology (ACR)/ European League Against Rheumatism (EULAR) 2012 criteria for RA, and patients with symptom duration up to one year (early arthritis) were invited to participate in the study. Nine patients with missing outcome data were excluded, as were another 8 patients who did not fulfil ACR/EULAR 2012 criteria for RA on a follow-up visit one year after their first visit (*n* = 83).

In order to form the control group, 350 subjects adjusted for the age and gender composition of the Estonian population in 2013 were randomly selected from a primary healthcare center practice list (total number of subjects: 1854). Cross-sectional data were gathered from September 2014 to March 2015. Invitations with introductory materials were mailed to 350 people. The primary healthcare center was contacted by a total number of 332 subjects for further instructions, and finally 330 people were recruited during the study period. Three subjects missed their study appointment, and six patients with missing outcome data were excluded (*n* = 321). The same control group was used in another population-based cross-sectional study conducted in the same geographic location [24]. Study procedures were performed after an overnight fast and before noon. An electronic scale was used to measure body weight in kilograms. Height was measured with a stadiometer, to the nearest 0.5 cm.

Body mass index (BMI) was calculated according to the standard formula: weight in kilograms divided by height in meters squared. Based on the World Health Organization (WHO) criteria, the following groups of BMI were formed: normal weight (BMI: ≤24.9 kg/m^2^), overweight (BMI: 25–29.9 kg/m^2^) and obese (BMI: ≥30 kg/m^2^) [30]. Both overweight and obese were classified as adiposity in analysis.

A Lunar Prodigy Dual-Energy X-Ray Absorptiometry (DXA) machine was used to separately measure arm, leg and whole body bone, lean and fat mass. A qualified and experienced technician performed all DXA measurements on all participating subjects.

The 30-Second Chair Stand Test (30-CST) and the Handgrip Strength Test (HST) were used to estimate and compare muscle strength. The 30-CST was used for assessing leg muscle function. While conducting the 30-CST, patients were asked to sit on a chair, with their spine straight and hands placed crossed across their chest, on opposite shoulders. On a signal, subjects rose at their own pace, to a full standing position, and then returned to seated position as many times as they were able. A 30 s trial was preceded by a practice attempt of 1–3 repetitions. The total number of unassisted full-stands during the 30 s time was considered the final chair-stand score [31].

The HST was used for assessing hand muscle function. Grip strength was measured with a calibrated Riester Dynatest dynamometer, in bars. One practice attempt was followed by three additional consecutive attempts, with pauses in between, of up to 60 s. The average value of three test trials of both hands was used for statistical analysis.

Blood samples included measuring vitamin D3, calcium, OPG and C-reactive protein (CRP). IL-6, TNFa, and IL-1b were evaluated, using Luminex’s xMAP technology. Anti-CCP, RF and erythrocyte sedimentation rate (ESR) were measured only in the arthritis group. An electrochemiluminescence assay was used with the cutoff value of 17 kU/L for anti-CCP positivity. RF was measured, using the immunoturbidimetric method, and it was assessed positive if RF value was >14 IU/mL. The ESR was evaluated by using a modified Westergren method.

To assess disease activity, the number of tender and swollen joints in the ERA group was evaluated (28 joint scores), and according to the standard formula, DAS28 scores were calculated, using CRP. According to the DAS28 score, patients were grouped as having low disease activity (DAS28 score <3.2), moderate disease activity (≥3.2 to ≤5.1) or high disease activity (>5.1) [32].

A 24 h dietary recall method was used for nutritional assessment, and data were analyzed with NutriData software developed by the Estonian National Institute for Health Development [33].

All data were tested for normality. The continuous data were presented as mean (±SD) if distributed normally, or else by median with 25% and 75% percentiles. Unpaired two-tailed Student’s *t*-test (for mean) and Mann–Whitney U test (for median) were used to make comparisons between ERA patients and controls.

Multiple linear regression with binary exposure variables was carried out, to assess bone, lean, fat mass of arms, as well as legs, and also muscle function mean difference between ERA (ERA = 1) and control group (control group = 0). Model was adjusted for age, gender, height and weight, as ERA patients differed from controls by age and gender. Multiple linear regression analysis was done to assess in ERA the association of smoking to arms and legs bone mass; the influence of CRP and the amount of consumed proteins to lean-mass changes. Smoking was chosen for bone analysis, as this can possibly have influence on bone changes [4,12]. CRP and proteins were used in lean-mass analysis, as they can possibly be associated with lean-mass changes [24].

All analyses were performed, using Statistica version 13.3 for Windows.

The study was approved by the Research Ethics Committee of the University of Tartu (population group study approval number 238/M-15, date of approval 16 July 2014), ERA group study approval: 221/M-9 (date of approval 17 December 2012). All subjects participating in the study signed written informed consent forms.

## 3. Results

### 3.1. General Characteristics of the Study Groups

ERA patients had significantly lower weight than controls. Moreover, ERA patients were older than controls. Smoking anamnesis had 33.7% (28) of ERA patients and 20.6% (66) of control subjects.

In total, 65.1% (54) of ERA patients were RF positive, and 71.1% (59) were anti-CCP positive (Table 1). A total of 58% (48) of ERA patients used disease-modifying anti-rheumatic drug (DMARDS): Methotrexate, Sulfasalazine and Hydroxychloroquine. Moreover, 71% (59) of ERA subjects used nonsteroidal anti-inflammatory drugs (NSAIDS). Glycocorticosteroids (GCS) were used by 27% (22) of ERA patients (Table 1).

### 3.2. Characteristics of Study Groups Inflammation and Bone Markers

The inflammation markers CRP, IL-6, TNFa and IL1-b were all significantly higher in the ERA group, as expected. OPG was almost two times higher in the ERA patients, as compared to control-group subjects (446 pg/mL versus 227 pg/mL, *p* < 0.0001; see Table 1). Median CRP and TNF-a were higher in male ERA patients, compared to ERA female patients, but the difference was not statistically significant.

### 3.3. Characteristics of Daily Food and Alcoholic Beverages of the Study Groups

In the nutritional status, ERA patients consumed less calories, proteins and fats, as compared with control subjects. Still, there was no significant difference in consumption of carbohydrates and alcohol between ERA patients and controls (Table 1).

### 3.4. Regression Analysis

We observed how the presence of ERA changes body structure (bone, lean and fat mass of arms, as well as legs) and function (mean handgrip, 30-CST), compared with control subjects. The multiple regression models adjusted for age, gender, height and weight revealed that in ERA subjects arm, leg and trunk bone-mass changes, due to the ERA disease, were not statistically significant (Table 2).

The results of regression analysis showed that the presence of ERA was associated with lower arm (b −304.6) and leg (b −943.4) lean mass compared with control subjects. ERA was also associated with lower mean handgrip in HST (b −0.08) and worse muscle strength of legs in the 30-CST (b −2.3), (Table 2).

The fat mass of arm was higher (b 220.0) in ERA compared with control group subjects, but there was no difference in leg fat mass (Table 2).

Additional regression analysis for only ERA subjects indicated that in ERA arm and leg bone mass changes were not associated with smoking (Table 3), RF positivity and OPG. The analysis also implied that, in ERA, the lean mass of arms and legs was negatively associated with CRP. The intake of proteins was not associated with lean-mass changes both in arms and legs (Table 3). Furthermore, smoking did not seem to have a relevant effect on muscle structural and functional changes, both on hands and legs.

## 4. Discussion

The aim of this study was to assess if there are structural and functional changes of hands and legs already in ERA, compared with the population-based control group. Additionally, we aimed to identify if the changes are symmetrical in hands and legs and if there are factors that associate with these changes. Our study confirmed that there are structural and functional changes of hands and legs already in ERA, compared with the population-based control group. In our ERA subjects (adjusted for age, gender, height and weight), arm and leg bone mass changes due to the ERA disease were not statistically significant. In ERA subjects, arm and leg bone mass changes were not associated with smoking, RF positivity and OPG. Smoking is a risk factor for bone-structure changes [34], but we assumed that, in ERA, the changes were not so evident yet, due to the short duration of RA disease.

RA patients have reported foot problems being a major concern affecting their lives [26,29]. Changes in forefoot anatomy have led to altered foot motion and increased pain during weight-bearing and walking [26]. Leg bone structure is often affected by RA. A recent study found that patients whose debut joint was in the foot had higher disease activity, higher dysfunction and also lower quality of life [35]. Still, the existing literature has little data about ERA leg changes, as studies tend to focus on hands. Furthermore, the most widely used scales evaluating arthritis activity do not include leg joints, and in some cases, patients only have MTP joint involvement instead of MCP joint arthritis. Therefore, it is important to imply that in ERA leg structure may change. While evaluating disease activity, it is very important to additionally assess leg joints, as there may already be relevant changes of leg structure in early phases of arthritis, as our study indicated.

Interestingly, we saw that the most evident changes were in limbs lean mass, and there was an especially prevalent reduction of leg lean mass in ERA patients. Accordingly, ERA patients had worse muscle function of hands in HST (*p* < 0.0001) and legs in the 30-S CST (*p* = 0.004), compared with control subjects. Similarly, it was found previously that muscle volume was lower in ERA compared with controls, indicating muscle atrophy in RA [36]. The results imply that the function of leg muscles in ERA patients may be possibly worse because they had lower leg lean mass, as compared to control subjects. The mean handgrip in ERA was lower, as can be expected due to lower arm lean mass and inflammation. In our ERA patients, smoking did not seem to have a relevant effect on muscle structural and functional changes in hands and legs, as previously believed [37]. It may be due to the short duration of RA and also because of the small size of study groups. As muscle strength has a significant role in preventing bone loss in RA [25], it is important to council ERA patients to be physically active.

Additionally, the fat mass of arm was higher in ERA, compared with control group subjects. This suggests that, in ERA, lean mass may decrease due to inflammation, possibly increasing fat proportion in the arm already in the early phase of RA.

As expected, our ERA patients had significantly higher inflammation markers, as compared with controls. As many pro-inflammatory cytokines (TNF-a, IL-6 and CRP) are produced by adipocytes, obese patients have elevated cytokine circulating plasma levels. Their levels seem to correlate with the amount of body fat and fall in response to weight loss [38]. TNF-a and IL-1 have a significant role in the inflammation-induced bone loss [39]. Still, in our ERA patients, the effect of inflammation on bone mass was not observed yet, probably due to the early course of RA disease. Unlike from previous data [37], possibly our ERA patients’ arm and leg lean mass was also lower, due to inflammation. The dissimilarity was probably due to the fact that the study was not conducted in ERA subjects, and also our study groups were different.

Previous data suggest that, in both genders, inflammation can be more severe when sex hormone levels decrease [40,41,42]. The level of testosterone in males can be decreased by inflammation and inversely correlates with IL-6 and CRP concentrations [41]. Estrogen deficiency in women can mediate an increase of IL-6 in serum and accelerate bone turnover [42]. Compared to ERA female patients, our male ERA subjects might possibly have a higher level of inflammation in serum, due to an age-related testosterone decrease.

Testosterone inhibits OPG production in men. In women, we see less of this effect [20], due to women’s lower testosterone levels. In obese women, serum concentrations of OPG were significantly lower, as compared to normal-weight controls, and further weight decreases caused additional decreases in OPG serum concentrations [43]. OPG was significantly higher in our ERA subjects, compared to controls. There was also a tendency that OPG was higher in more active arthritis, indicating the effects of inflammation. Interestingly, OPG levels were higher in our female ERA subjects, possibly due to the impact of fat tissue, as their total body fat mass was higher compared to males. Still, in our ERA subjects, arm and leg bone-mass changes were not associated with OPG, possibly due to the short duration of RA.

In addition to hormonal changes, it has been previously suggested that, as a part of diet, daily intake of monounsaturated fatty acids may lower disease activity in RA patients [44], and therefore possibly help diminish the appearance of RA-specific changes. Our ERA patients consumed fewer calories, proteins and fats, compared to control subjects. Inflammation due to early arthritis can possibly cause a loss of appetite, and therefore lower consumption of calories, including proteins. Previous studies have indicated that low protein intake was related to accelerated decreases in muscle strength in older persons with a pro-inflammatory state [45] and lower appendicular lean mass in ERA [24]. Our data indicated that the lowering of lean mass of arm and leg in ERA was not associated with a lower intake of proteins. We can speculate that the contrast may be due to the short duration of ERA disease and different statistical analysis.

We can consider it a limitation that we did not assess the level of sex hormones, which can possibly alter the course of disease. Our study was also limited by small study groups, but this was due to the small total size of the Estonian population.

## 5. Conclusions

The results of the conducted study indicated that there are structural and functional changes of hands and legs already in ERA, compared with the population-based control group. The changes of hands and legs are different in ERA. ERA patients had lower lean mass of arm and leg and, accordingly, decreased muscle function. The lowering of lean mass of arm and leg in ERA was associated with the elevation of CRP. ERA patients also had higher fat mass of arm. It seems that lifestyle factors do not affect hands and legs in ERA. Arthritis involvement can vary among people, so it is therefore important in ERA to assess hands and legs in parallel, as the structural changes differ. This knowledge may alter the evaluation of disease activity, affect treatment strategy and perhaps someday help work out a new score for better assessing ERA patients.

## Figures and Tables

**Table 1:** Characteristics of the study groups.

	ERA Patients	Controls	*p*-Value
	(*n* = 83)	(*n* = 321)	
General characteristics			
Female gender, *n* (%)	60 (72)	175 (54)	0.004
Age, years	52.7 (15.7)	47.9 (16.5)	0.018
Weight, kg	74.7 (14.8)	80.2 (17.6)	0.009
Height, cm	166 (9)	171 (10)	<0.0001
BMI (kg/m^2^)	27.2 (5.6)	27.2 (5.3)	0.960
BMI groups, *n* (%)			
Normal	32 (38.6)	123 (38.3)	
Overweight	30 (36.1)	104 (32.4)	0.723
Obese	21 (25.3)	94 (29.3)	
Smoking (ever), *n* (%)	28 (33.7)	66 (20.6)	0.014
Arm structure			
Arm bone mass	355 (92)	393 (116)	0.005
Arm lean mass	4893 (1307)	6018 (2042)	<0.0001
Arm fat mass	2701 (1230)	2420 (1165)	0.053
Leg structure			
Leg bone mass	1042 (201)	1149 (262)	0.0005
Leg lean mass	14019 (2942)	16798 (4135)	<0.0001
Leg fat mass	8913 (3850)	8235 (3864)	0.155
Function			
Mean handgrip	0.28 (0.20)	0.46 (0.24)	<0.0001
30-CST	14.0 (6.1)	17.4 (7.7)	0.0002
Daily consumed food and alcoholic beverages (last 24 h)			
Calories (kcal)	1507 (625)	1832 (786)	0.0005
Carbohydrates (g)	196 (97)	210 (91)	0.223
Proteins (g)	53 (38–69)	73 (53–99)	<0.0001
Fats (g)	53(40–72)	66 (46–97)	0.002
Alcohol (g)	6.3 (1.5)	33.9 (35.0)	0.181
Inflammation and bone markers, disease activity			
IL-6 pg/ml	2.9 (0–19.0)	0 (0–0)	<0.0001
TNFa pg/ml	2.2 (1.6–3.0)	1.8 (1.4–2.3)	<0.0001
IL-1b pg/mL	0.1 (0–1.0)	0 (0–0)	<0.0001
CRP mg/L	4.0 (1.6–18.0)	0.9 (0.2–2.5)	<0.0001
ESR mm/h	20.7 (20.4)		
male mean	31.6 (23.8)		
female mean	16.5 (17.4)		
OPG pg/mL	446 (334–658)	227 (177–299)	<0.0001
Ca mmol/L	2.4 (0.3)	2.3 (0.1)	0.0005
25 (OH) Vitamin D nmol/L	55 (22)	41 (18)	<0.0001
Rheumatoid arthritis specific characteristics			
Time from first RA			
symptoms, days	88 (48–245)		
RF positivity, *n* (%)	54 (65.1)		
Anti-CCP positivity, *n* (%)	59 (71.1)		
DAS28 score (CRP)	3.9 (1.3)		
male mean	4.2 (1.3)		
female mean	3.8 (1.2)		

BMI, body mass index; ERA, early rheumatoid arthritis; CRP, C-reactive protein; ESR, erythrocyte sedimentation rate; 30-CST, 30-Second Chair Stand Test; OPG, osteoprotegerin; RF, rheumatoid factor. RF positive, when RF > 14 IU/mL. Anti-CCP: anti-citrullinated protein antibodies. Anti-CCP positive, when ≥17 kU/L. Values in table represent mean (SD) or median (with interquartile range). Used units: mass (g), function (bars).

**Table 2:** Mean estimated difference of body structure and function in ERA, compared to controls *.

Dependent Variable	Regression Coefficient (b)	Standard Error	*p*-Value
Arm structure			
Arm bone mass	9.6	6.5	0.144
Arm lean mass	−304.6	98.8	0.002
Arm fat mass	220	62.0	0.0005
Leg structure			
Leg bone mass	7.5	14.0	0.618
Leg lean mass	−943.4	184.9	<0.0001
Leg fat mass	417.5	241.0	0.080
Function			
Mean handgrip	−0.08	0.02	<0.0001
30-CST	−2.3	0.8	0.004

* Multiple regression models adjusted for age, gender, height and weight; 30-CST, 30-Second Chair Stand Test.

**Table 3:** Summary of regression analysis for variables predicting ERA-specific body structural changes *.

Dependent Variable	Predictor Variable	Regression Coefficient (b)	Standard Error	*p*-Value
Arm bone mass	Smoking	14.7	12.3	0.237
Arm lean mass	CRP	−11.13	3.5	0.0002
	Proteins	−0.6	2.7	0.824
Leg bone mass	Smoking	31.0	25.4	0.226
Leg lean mass	CRP	−18.6	9.2	0.047
	Proteins	6.0	7.1	0.403

* Different multiple regression models adjusted for age, gender, height and weight.

## Data Availability

The datasets used and analyzed during the current study are available from the corresponding author on reasonable request.

## References

[1] Scott D.L., Wolfe F., Huizinga T.W. (2010). Rheumatoid arthritis. Lancet.

[2] Bultink I.E., Vis M., van der Horst-Bruinsma I.E., Lems W.F. (2012). Inflammatory rheumatic disorders and bone. Curr. Rheumatol. Rep..

[3] Otsa K., Tammaru M., Vorobjov S., Esko M., Pärsik E., Lang K. (2013). The prevalence of rheumatoid arthritis in Estonia: An estimate based on rheumatology patients’ database. Rheumatol. Int..

[4] Sugiyama D., Nishimura K., Tamaki K., Tsuji G., Nakazawa T., Morinobu A., Kumagai S. (2009). Impact of smoking as a risk factor for developing rheumatoid arthritis: A meta-analysis of observational studies. Ann. Rheum. Dis..

[5] Chang K., Yang S.M., Kim S.H., Han K.H., Park S.J., Shin J.I. (2014). Smoking and rheumatoid arthritis. Int. J. Mol. Sci..

[6] Saevarsdottir S., Rezaei H., Geborek P., Petersson I., Ernestam S., Albertsson K., Forslind K., van Vollenhoven R.F. (2015). SWEFOT study group. Current smoking status is a strong predictor of radiographic progression in early rheumatoid arthritis: Results from the SWEFOT trial. Ann. Rheum. Dis..

[7] De Rooy D.P.C., Nies J.A.B.V., Kapetanovic M.C., Kristjansdottir H., E Andersson M.L., Forslind K., Van Der Heijde D.M.F.M., Gregersen P.K., Lindqvist E., Huizinga T.W.J. (2014). Smoking as a risk factor for the radiological severity of rheumatoid arthritis: A study on six cohorts. Ann. Rheum. Dis..

[8] Kilic G., Ozgocmen S. (2015). Hand bone mass in rheumatoid arthritis: A review of the literature. World J. Orthop..

[9] Njeh C.F., Genant H.K. (2000). Bone loss. Quantitative imaging techniques for assessing bone mass in rheumatoid arthritis. Arthritis Res..

[10] Haugeberg G., Green M.J., Quinn M.A., Marzo-Ortega H., Proudman S., Karim Z., Wakefield R.J., Conaghan P.G., Stewart S., Emery P. (2006). Hand bone loss in early undifferentiated arthritis: Evaluating bone mineral density loss before the development of rheumatoid arthritis. Ann. Rheum Dis..

[11] Schett G., Redlich K., Smolen J.S. (2003). The role of osteoprotegerin in arthritis. Arthritis Res. Ther..

[12] Hill C.L., Schultz C.G., Wu R., Chatterton B.E., Cleland L.G. (2010). Measurement of hand bone mineral density in early rheumatoid arthritis using dual energy X-ray absorptiometry. Int. J. Rheum Dis..

[13] Daragon A., Krzanowska K., Vittecoq O., Ménard J.F., Hau I., Jouen-Beades F., Lesage C., Bertho J.M., Tron F., Le Loët X. (2001). Prospective X-ray densitometry and ultrasonography study of the hand bones of patients with rheumatoid arthritis of recent onset. Joint Bone Spine.

[14] Van der Heijde D.M., van Leeuwen M.A., van Riel P.L., van de Putte L.B. (1995). Radiographic progression on radiographs of hands and feet during the first 3 years of rheumatoid arthritis measured according to Sharp’s method (van der Heijde modification). J. Rheumatol..

[15] Nell V.P., Machold K.P., Eberl G., Stamm T.A., Uffmann M., Smolen J.S. (2004). Benefit of very early referral and very early therapy with disease-modifying anti-rheumatic drugs in patients with early rheumatoid arthritis. Rheumatology.

[16] Takayanagi H. (2009). Osteoimmunology and the effects of the immune system on bone. Review. Nat. Rev. Rheumatol..

[17] Lacey D.L., Timms E., Tan H.L., Kelley M.J., Dunstan C.R., Burgess T., Elliott R., Colombero A., Elliott G., Scully S. (1998). Osteoprotegerin ligand is a cytokine that regulates osteoclast differentiation and activation. Cell.

[18] Geusens P.P., Lems W.F. (2011). Osteoimmunology and osteoporosis. Arthritis Res. Ther..

[19] Schett G., Saag K.G., Bijlsma J.W.J. (2010). From bone biology to clinical outcome: State of the art and future perspectives. Ann. Rheum Dis..

[20] Bianchi V.E. (2018). The Anti-Inflammatory Effects of Testosterone. J. Endocr. Soc..

[21] Perez de Ciriza C., Moreno M., Restituto P., Bastarrika G., Simón I., Colina I., Varo N. (2014). Circulating osteoprotegerin is increased in the metabolic syndrome and associates with subclinical atherosclerosis and coronary arterial calcification. Clin. Biochem..

[22] Hofbauer L.C., Hicok K.C., Chen D., Khosla S. (2002). Regulation of osteoprotegerin production by androgens and anti-androgens in human osteoblastic lineage cells. Eur J. Endocrinol..

[23] Pathak J.L., Bravenboer N., Luyten F.P., Verschueren P., Lems W.F., Klein-Nulend J., Bakker A.D. (2015). Mechanical Loading Reduces Inflammation-Induced Human Osteocyte-to-Osteoclast Communication. Calcif. Tissue Int..

[24] Müller R., Kull M., Põlluste K., Valner A., Lember M., Kallikorm R. (2019). Factors Associated with Low Lean Mass in Early Rheumatoid Arthritis: A Cross-Sectional Study. Medicina.

[25] Liphardt A.M., Windahl S.H., Sehic E., Hannemann N., Gustafsson K.L., Bozec A., Schett G., Engdahl C. (2020). Changes in mechanical loading affect arthritis-induced bone loss in mice. Bone.

[26] Stolt M., Suhonen R., Leino-Kilpi H. (2017). Foot health in patients with rheumatoid arthritis-a scoping review. Rheumatol. Int..

[27] Bálint G.P., Korda J., Hangody L., Bálint P.V. (2003). Regional musculoskeletal conditions: Foot and ankle disorders. Best Pract. Res. Clin. Rheumatol..

[28] Otter S.J., Lucas K., Springett K., Moore A., Davies K., Cheek L., Young A., Walker-Bone K. (2010). Foot pain in rheumatoid arthritis prevalence, risk factors and management: An epidemiological study. Clin. Rheumatol..

[29] Williams A.E., Graham A.S. (2012). ‘My feet—Visible, but ignored…’ A qualitative study of foot care for people with rheumatoid arthritis. Clin. Rehabil..

[30] World Health Organization (1997). Obesity: Preventing and Managing the Global Epidemic.

[31] Unver B., Kalkan S., Yuksel E., Kahraman T., Karatosun V. (2015). Reliability of the 50-foot walk test and 30-sec chair stand test in total knee arthroplasty. Acta Ortop. Bras..

[32] Anderson J., Caplan L., Yazdany J., Robbins M.L., Neogi T., Michaud K., Saag K.G., O’dell J.R., Kazi S. (2012). Rheumatoid Arthritis Disease Activity Measures: American College of Rheumatology Recommendations for Use in Clinical Practice. Arthritis Care Res..

[33] NutriData Software Homepage. http://tap.nutridata.ee/.

[34] Llorente I., García-Castañeda N., Valero C., González-Álvaro I., Castañeda S. (2020). Osteoporosis in Rheumatoid Arthritis: Dangerous Liaisons. Front. Med..

[35] Yano K., Ikari K., Inoue E., Sakuma Y., Mochizuki T., Koenuma N., Tobimatsu H., Tanaka E., Taniguchi A., Okazaki K. (2018). Features of patients with rheumatoid arthritis whose debut joint is a foot or ankle joint: A 5,479-case study from the IORRA cohort. PLoS ONE.

[36] Farrow M., Biglands J., Tanner S., Hensor E.M.A., Buch M.H., Emery P., Tan A.L. (2020). Muscle deterioration due to rheumatoid arthritis: Assessment by quantitative MRI and strength testing. Rheumatology.

[37] Baker J.F., Mostoufi-Moab S., Long J., Taratuta E., Leonard M.B., Zemel B. (2019). Low Muscle Density is Associated with Deteriorations in Muscle Strength and Physical Functioning in Rheumatoid Arthritis. Arthritis Care Res..

[38] Vachharajani V., Granger D.N. (2009). Adipose tissue: A motor for the inflammation associated with obesity. IUBMB Life..

[39] Fardellone P., Salawati E., Le Monnier L., Goëb V. (2020). Bone Loss, Osteoporosis, and Fractures in Patients with Rheumatoid Arthritis: A Review. J. Clin. Med..

[40] Chen C.W., Jian C.Y., Lin P.H., Chen C.C., Lieu F.K., Soong C., Hsieh C.C., Wan C.Y., Idova G., Hu S. (2016). Role of testosterone in regulating induction of TNF-a in rat spleen via ERK signaling pathway. Steroids.

[41] Kapoor D., Clarke S., Stanworth R., Channer K.S., Jones T.H. (2007). The effect of testosterone replacement therapy on adipocytokines and C-reactive protein in hypogonadal men with type 2 diabetes. Eur. J. Endocrinol..

[42] Mysliwiec J., Zbucki R., Nikolajuk A., Mysliwiec P., Taranta A., Kaminski K., Bondyra Z., Dadan J., Gorska M., Win-nicka M.M. (2010). Role of interleukin-6 on RANKL-RANK/osteoprotegerin system in hypothyroid ovariectomized mice. Folia Histochem. Cytobiol..

[43] Holecki M., Zahorska-Markiewicz B., Janowska J., Nieszporek T., Wojaczynska-Stanek K., Zak-Gołab A., Wiecek A. (2007). The influence of weight loss on serum osteoprotegerin concentration in obese perimenopausal women. Obesity.

[44] Matsumoto Y., Sugioka Y., Tada M., Okano T., Mamoto K., Inui K., Habu D., Koike T. (2018). Monounsaturated fatty acids might be key factors in the Mediterranean diet that suppress rheumatoid arthritis disease activity: The TOMORROW study. Clin. Nutr..

[45] Bartali B., Frongillo E.A., Stipanuk M.H., Bandinelli S., Salvini S., Palli D., Morais J.A., Volpato S., Guralnik J.M., Fer-rucci L. (2012). Protein intake and muscle strength in older persons: Does inflammation matter?. J. Am. Geriatr. Soc..

